# *Rosa laevigata* Michx. Polysaccharide Ameliorates Diabetic Nephropathy in Mice through Inhibiting Ferroptosis and PI3K/AKT Pathway-Mediated Apoptosis and Modulating Tryptophan Metabolism

**DOI:** 10.1155/2023/9164883

**Published:** 2023-10-05

**Authors:** Tianyu Zhang, Wenjuan Sun, Lixin Wang, Hui Zhang, Yuansong Wang, Baochao Pan, Hanzhou Li, Ziang Ma, Kai Xu, Huantian Cui, Shuquan Lv

**Affiliations:** ^1^Cangzhou Hospital of Integrated Traditional Chinese Medicine and Western Medicine of Hebei Province Affiliated to Hebei University of Chinese Medicine, Cangzhou, China; ^2^Graduate School of Chengde Medical University, Chengde, China; ^3^Graduate School of Hebei University of Chinese Medicine, Shijiazhuang, China; ^4^Yunnan University of Traditional Chinese Medicine, Kunming, China

## Abstract

Diabetic nephropathy (DN) is a metabolic disease wherein chronic hyperglycemia triggers various renal cell dysfunctions, eventually leading to progressive kidney failure. *Rosa laevigata* Michx. is a traditional Chinese herbal medicine. Many studies have confirmed its antioxidative, anti-inflammatory, and renoprotective effects. However, the effects and mechanisms of *Rosa laevigata* Michx. polysaccharide (RLP) in DN remain unclear. In this study, a DN mouse model was established to investigate the therapeutic effect of RLP on DN mice. Then, nontargeted metabolomics was used to analyze the potential mechanism of RLP in the treatment of DN. Finally, the effects of RLP on ferroptosis and the PI3K/AKT pathway were investigated. The results demonstrated that RLP effectively alleviated renal injury and reduced inflammation and oxidative stress in the kidney. In addition, nontargeted metabolomic analysis indicated that RLP could modulate riboflavin metabolism and tryptophan metabolism in DN mice. Notably, ferroptosis and PI3K/AKT pathway-mediated apoptosis in the kidney were also ameliorated following RLP treatment. In conclusion, this study confirmed that RLP had a significant therapeutic effect on DN mice. Furthermore, RLP treatment modulated tryptophan metabolism and inhibited ferroptosis and PI3K/AKT pathway-mediated apoptosis in the kidney.

## 1. Introduction

Diabetic nephropathy (DN) is a metabolic disease wherein chronic hyperglycemia triggers various renal cell dysfunctions, eventually leading to progressive kidney failure [1]. The pathophysiological mechanism of DN is complex, with factors such as metabolic abnormalities, hemodynamic changes, inflammation, and autophagy disorders reportedly contributing to the onset of DN [2]. Although some therapeutic approaches are available at present, none of these approaches are effective in reversing the progression of DN [3]. Therefore, the development of novel drugs is crucial to delay and block the progression of DN.

Many natural products exert a protective effect against DN [4].For example, glabridin, a bioactive component of *Glycyrrhiza glabra* L., can alleviate DN by inhibiting ferroptosis and the VEGF/AKT/ERK pathway [5]. Salidroside, an active ingredient of *Rhodiola crenulata* (Hook.f. & Thomson) H. Ohba, can improve DN by regulating mitochondrial biogenesis mediated by the Sirt1/PGC-1*α* pathway [6]. Bruceine A, a steroid compound extracted from *Brucea javanica* (L.) Merr., can selectively bind to the conserved carbohydrate recognition domain of galectin-1 and disrupt the interaction between galectin-1 and RACK1, thereby exerting a significant protective effect against DN [7]. Recently, polysaccharides derived from natural herbs have been demonstrated with renoprotective potentials in DN. *Bupleurum chinense* polysaccharides had protective effects on DN mice through modulating the gut microbiota and inhibiting inflammation [8]. *Ganoderma lucidum* polysaccharides could ameliorate DN via inhibiting apoptosis and inflammation and activating autophagy [9]. *Grifola frondosa* polysaccharides had the ability to control blood glucose and alleviate renal fibrosis [10]. Therefore, evaluating the effects and elucidating the mechanisms of herbal polysaccharides on DN is essential for developing novel drugs.

*Rosa laevigata* Michx. is a traditional Chinese herbal medicine. Numerous studies have confirmed its antioxidant, anti-inflammatory, and renoprotective effects [11]. The total flavonoids of *Rosa laevigata* Michx. can improve oxidative stress and renal ischemia-reperfusion injury [12, 13]. However, the effects and mechanisms of *Rosa laevigata* Michx. polysaccharide (RLP) in DN remain unclear. In this study, a DN mouse model was established to investigate the therapeutic effect of RLP on DN mice. Then, nontargeted metabolomics was used to investigate the potential mechanism underlying the therapeutic effects of RLP in DN. Finally, the effects of RLP on ferroptosis and the PI3K/AKT pathway were investigated.

## 2. Methods

### 2.1. Animals and Materials

Sixty specific-pathogen-free-grade male C57BL/6 mice weighing 20–22 g were purchased from Beijing Huafukang Biotechnology Co., Ltd. Animal license number: SCXK (Beijing) 2019-0008. Each cage housed 5 mice at room temperature (21 ± 2°C) with *ad libitum*. All animal experiments were approved by the ethics committee of the Cangzhou Hospital of Integrated Traditional Chinese Medicine and Western Medicine and performed in accordance with the *Guide for the Care and Use of Laboratory Animals*. Details of materials and reagents are included in the supplementary materials (available here).

### 2.2. Animal Experiment

After 1 week of acclimatization, 50 mice were randomly chosen to be fed a high-sugar and high-fat diet (HFD, 21% fat, 34% sucrose, 0.15% cholesterol, and 44.85% normal chow) for 8 weeks as experimental group. The remaining 10 mice took normal chow as a control group. At the end of the eighth week, after fasting for 12 hours, mice in the experimental group were intraperitoneally injected with 30 mg/kg of streptozotocin (STZ), and those in the control group were intraperitoneally injected with an equivalent volume of 1% sodium citrate buffer. After 72 hours, the random blood glucose level was measured. Type 2 diabetes mellitus (T2DM) was defined as blood glucose level of ≥16.7 mM. Thereafter, feeding was continued, and the 24 h urine protein content of mice was assessed every week. A random blood glucose level of ≥16.7 mM and a 24-h urine protein content ≥20 mg was used as the standard for the DN model [14].

After modeling, mice in an experimental group were randomly assigned to the model group, irbesartan (IRB) group, RLP low-dose group (L-RLP), RLP medium-dose group (M-RLP), or RLP high-dose group (H-RLP), with 10 mice in each group. Mice in the control and model groups were administered with 0.2 mL/d of normal saline, while those in the IRB group were administered with 50 mg/kg/d of IRB [15]. The L-RLP, M-RLP, and H-RLP groups were administered with 20, 40, and 80 mg/kg/d of RLP, respectively, for 4 consecutive weeks. The fasting blood glucose (FBG) level and body weight of mice in each group were measured every week. After the treatment, 24 h urine samples were collected from mice in each group using metabolic cages. Blood samples were also collected by drawing blood from the inner canthus of the eye. After euthanasia, the left kidney of mice was collected and fixed in 4% paraformaldehyde, while the right kidney was cryopreserved.

### 2.3. Determination of Biochemical Indices

The 24 h urine sample was centrifuged at 4000 g for 10 min to separate the supernatant. The 24 h protein content in the urine sample was detected according to the manufacturer's instructions of assay kit. The blood samples were centrifuged at 400 g for 15 min to collect the serum. Creatinine (Cr) and blood urea nitrogen (BUN) levels in the serum of mice in each group were determined following the manufacturer's instructions of assay kits. Partially frozen kidney tissues and normal saline were mixed at a ratio of 1 : 9 and homogenized. The supernatant was collected after centrifugation at 400 g for 15 min. Oxidative stress-related indicators, including superoxide dismutase (SOD) and glutathione peroxidase (GSH-Px) activities, as well as malondialdehyde (MDA) and reactive oxygen species (ROS) level, were detected according to the manufacturer's instructions of the relevant kits. Meanwhile, the total protein concentration in the tissue homogenate was determined to normalize the sample data.

### 2.4. Pathological Staining

After fixing for 24 hours, kidney tissue specimens were subjected to graded ethanol dehydration and slices into 3 *μ*m paraffin sections for hematoxylin and eosin (H&E) staining and periodic acid-Schiff (PAS) staining. Histopathological changes were analyzed under an optical microscope.

### 2.5. Enzyme-Linked Immunosorbent Assay (ELISA)

The levels of interleukin- (IL-) 6, IL-1*β*, tumor necrosis factor- (TNF-) *α*, and 4-hydroxynonenal (4-HNE) in the kidney tissue homogenate were determined using one-step ELISA. According to the manufacturer's instructions, the samples were added to the antibody-coated ELISA plate, and a standard curve was plotted using the standards provided in the kit. After incubation, the plate was washed, and the enzyme-labeled secondary antibody was added. Subsequently, the plate was washed after incubation. After adding the chromogenic solution, absorbance was read at 405 nm using a microplate reader. The levels of IL-6, IL-1*β*, TNF-*α*, and 4-HNE were calculated using the standard curves.

### 2.6. Metabolomic Analysis

Frozen kidney tissue samples (100 mg) were grounded in liquid nitrogen. Subsequently, 500 *μ*L of 80% methanol was added, and the tube was vortexed, oscillated, and cooled in an ice bath for 5 minutes. The mixture was then centrifuged at 4°C (15,000 g, 20 min). The supernatant was diluted with a solution containing 53% methanol and ultrapure water and centrifuged again at 4°C (15,000 g, 20 min) to separate the supernatant. To make quality control samples, an equal 20 *μ*L aliquot of each sample was drawn and mixed together. The detailed conditions for liquid chromatography-mass spectrometry and the steps for data acquisition, processing, and analysis are provided in supplementary material.

### 2.7. Real-Time Quantitative PCR (RT-qPCR)

Routine procedures were performed, as described previously [16]. Total RNA was extracted from kidney tissues using the TRIzol reagent and was reverse transcribed into cDNA using a commercial kit. Gene expression analysis was performed using a real-time PCR detection system and a SuperReal PreMix Plus kit. The mRNA expression level of the target gene relative to the housekeeping gene *Actb* expression level was calculated using the 2^-*ΔΔ*CT^ method. The primer sequences for *Actb*, *Kmo*, *Kynu*, *Tph1*, and *Aanat* are included in the supplementary material.

### 2.8. Western Blot

Total protein extraction from kidney tissues was performed using radioimmunoprecipitation-assay lysis buffer. The bicinchoninic acid assay kit was used to determine the protein concentrations and normalize the samples. Then, protein loading buffer was added to the sample and mixed well. The mixture was subjected to 95°C for 5 minutes to denature the proteins. Protein samples were resolved by electrophoresis using SDS-PAGE and transferred to polyvinylidene fluoride membranes. After blocking with 10% skimmed milk for 1 hour, the membrane was incubated overnight at 4°C with the primary antibody for KMO, AANAT, TPH1, AANAT, transferrin, STEAP3, SLC7A11, SLC3A2, GPX4, GCLC, or ACTB. After incubation, the membrane was washed three times with TBST and incubated with the secondary antibody for 1 hour. After washing with TBST for three times, enhanced chemiluminescence (ECL) substrates were added, and the membrane was developed and exposed with a chemiluminescent detection system to obtain the western blot bands. ImageJ was used to quantify the relative protein levels.

### 2.9. Statistical Methods

SPSS Statistics 17.0 was used for data analysis. T-test was performed for measurement data following normal distribution. Data are expressed as mean ± standard deviation. Intergroup comparison was performed using a *t-test.P* < 0.05 was considered statistically significant.

## 3. Results

### 3.1. Therapeutic Effects of RLP in DN Mice

**Figure 1:**
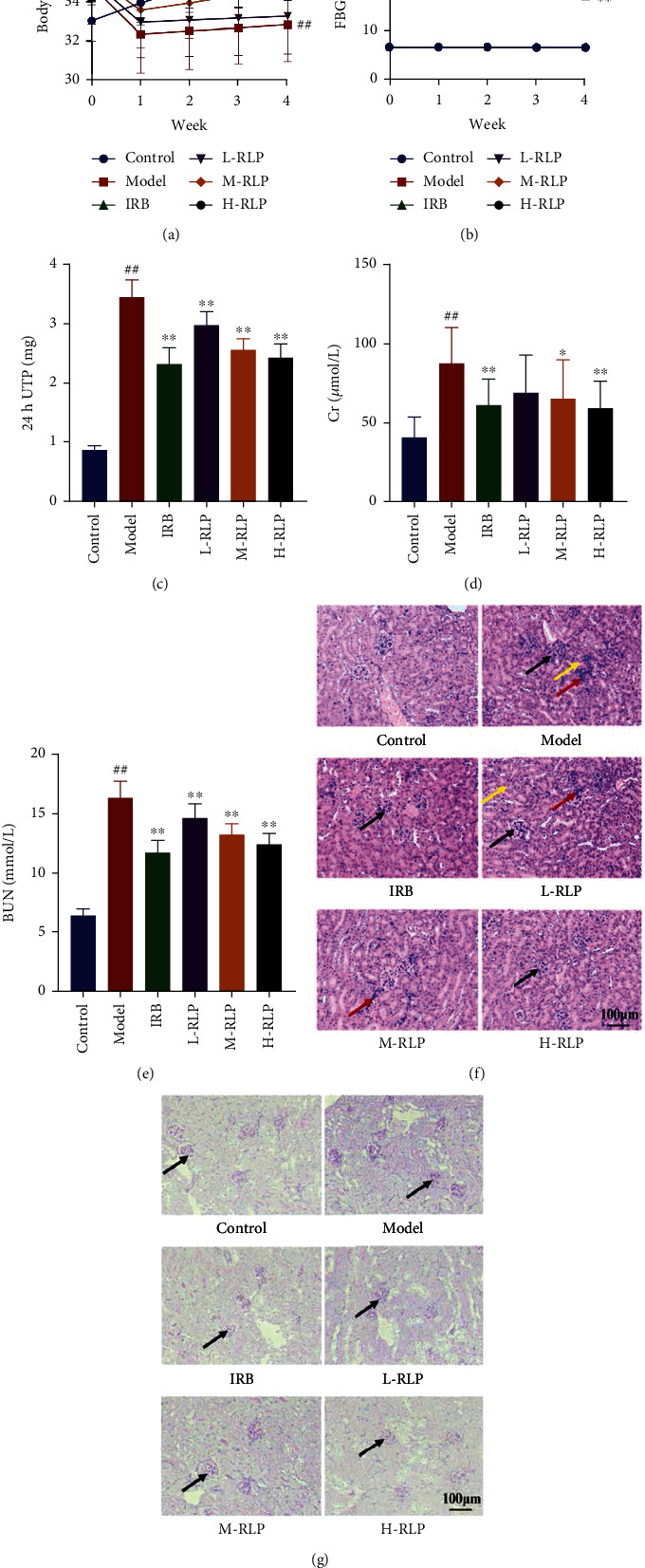
RLP has a therapeutic effect on DN mice. After 42 days of treatment, all mice were euthanized. The urine, blood, and kidney samples were collected and analysed. The effects of RLP on the change curves of body weight (a) and FBG (b) in each group were examined. The changes in kidney function, such as 24 h UTP (c), Cr (d), and BUN (e), after RLP treatment were also evaluated. Kidney tissues were subjected to HE staining (f) and PAS staining (g) for pathological analysis. In HE staining, black arrows indicated the thickening of the glomerular basement membrane, yellow arrows indicated the cellular swelling of renal tubular epithelial cells, and red arrows indicated the infiltration of inflammatory cells. In PAS staining, black arrows indicated the glomerulus (100×). Control, model, IRB, L-RLP, M-RLP, and H-RLP (*n* = 10 per group) groups. Data are presented as the mean ± standard deviation. ##*P* < 0.01 as compared to the control group; ^∗^*P* < 0.05 as compared to the model group; ^∗∗^*P* < 0.01 as compared to the model group.

Significant differences were observed in the trends of the body weight curve and the FBG curve during the 4-week treatment period. Compared with that noted in the control group, the weight of the mice in the model group decreased significantly, while mice treated with IRB, M-RLP, and H-RLP showed significantly delayed weight loss (Figure 1(a)). Compared with that noted in the control group, fasting blood glucose level was significantly increased in the model group, while significantly controlled blood glucose levels were noted in both IRB and RLP treatment groups (Figure 1(b)). The renal function test results showed that, compared with that noted in the control group, significant increases were noted in the serum levels of Cr and BUN, as well as the 24 h urine protein level in the model group, while these indicators improved to varying degrees in the RLP treatment groups (Figures 1(c)–1(e)). Importantly, the most significant effect was seen in the H-RLP group. These indicators also showed significant improvement in the IRB group. Considering the H&E and PAS staining results, compared with that noted in the control group, severe pathological damage, including hyperplasia of the mesangial matrix, thickening of the glomerular basement membrane, cellular swelling of renal tubular epithelial cell, and infiltration of inflammatory cells, was seen in the kidneys of mice in the model group. In contrast, the renal condition improved in the IRB and RLP treatment groups (Figure 1(d)).

### 3.2. Effects of RLP on the Oxidative Stress and Inflammatory Factors in DN Mice

**Figure 2:**
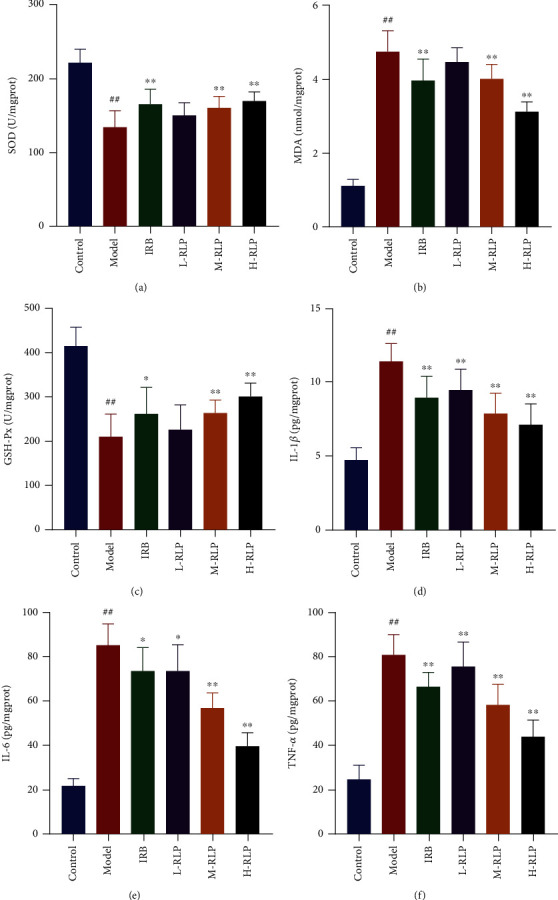
RLP has antioxidative and anti-inflammatory effects on DN mice. To investigate the anti-inflammatory and antioxidative effects of RLP, the study analysed the impact of RLP treatment on oxidative-related factors and proinflammatory cytokines in the kidney of DN mice. RLP enhanced the activities of SOD (a) and GSH-Px (c) and reduced the level of MDA (b) in the kidney, indicating an improvement in oxidative stress. Moreover, RLP administration to DN mice resulted in a decrease in the levels of IL-1*β* (d), IL-6 (e), and TNF-*α* (f) in the kidney, suggesting a reduction in inflammation.

In comparison to the control group, the model group exhibited decreased activities of SOD and GSH-PX, along with a significant increase in the MDA level, suggesting that the kidneys of DN mice were subjected to serious oxidative stress (Figures 2(a)–2(c)). Moreover, compared with that noted in the model group, the SOD and GSH-Px activities of mice in the IRB and H-RLP groups were increased, while MDA concentration was decreased. These suggested RLP significantly alleviated the oxidative stress in the kidneys of DN mice. The levels of IL-6, IL-1*β*, and TNF-*α* were significantly increased in the model group compared to the control group. However, these inflammatory factors were significantly alleviated in the IRB and RLP groups. (Figures 2(d)–2(f)). Furthermore, the effect was particularly significant in the H-RLP group. This indicated that RLP treatment significantly improved the inflammation in the kidneys of DN mice.

### 3.3. Effects of RLP on Metabolites in Kidney Tissues of DN Mice

**Figure 3:**
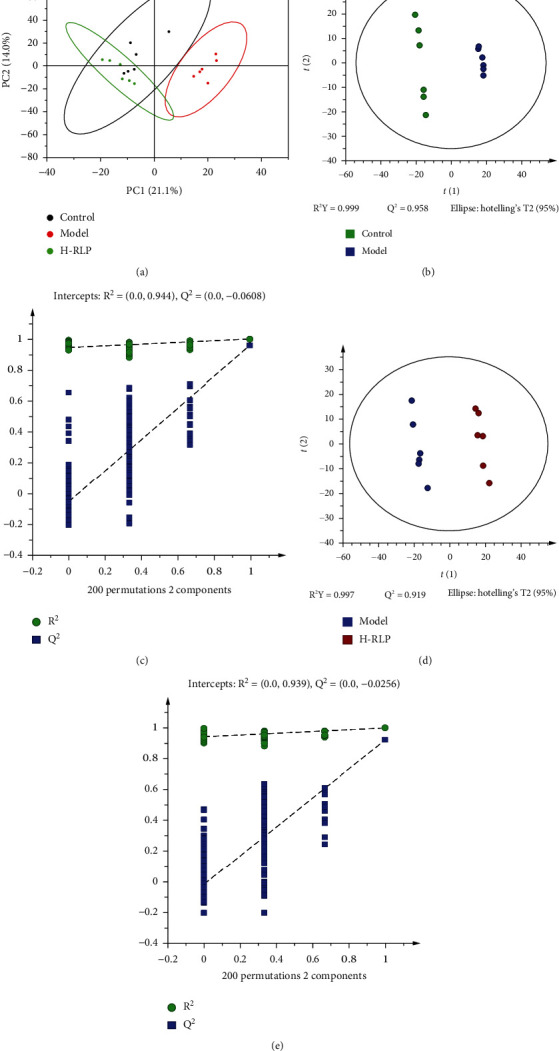
RLP alters the metabolites of the kidney in DN mice. To evaluate the effects of RLP on DN mice, multivariate statistical analysis and pathway analysis were conducted. (a) Score plots of PCA showed the difference among control, model, and RLP groups. (b–e) Score plots and permutation tests of PLS-DA were performed to compare the control and model groups (b, c) as well as the model and RLP groups (d, e).

In the principal component analysis (PCA) score plot of the control group, model group, and H-RLP group, each group was clustered together. These three groups were significantly separated, indicating that H-RLP significantly changed the abnormality of mouse kidney metabolites caused by DN (Figure 3(a)). A prediction model was established using partial least squares-discriminant analysis (PLS-DA). The PLS-DA score plot demonstrated a clear separation between the control group and model group, as well as between the model group and H-RLP group, with both R^2^Y being greater than 0.9 and *Q*^2^ being less than zero, indicating that the model did not appear to be overfitting, confirming the high explanatory power of the results (Figures 3(b)–3(e)).

**Figure 4:**
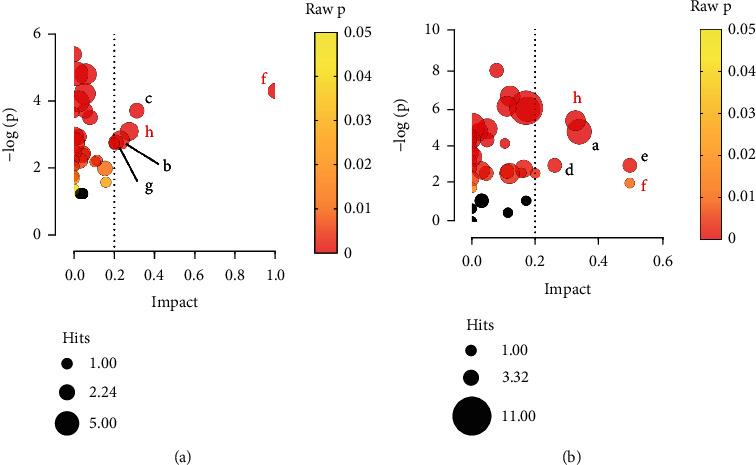
RLP alters the metabolic pathways of the kidney in DN mice. Pathway analysis results showed the differential metabolism pathways between the control and model groups (a) as well as model and RLP groups (b). Common pathways are marked in red. Black bubbles indicate pathways with *P* ≥ 0.05. Name of pathways: a: alanine, aspartate, and glutamate metabolism; b: arginine biosynthesis; c: histidine metabolism; d: phenylalanine metabolism; e: phenylalanine, tyrosine, and tryptophan biosynthesis; f: riboflavin metabolism; g: starch and sucrose metabolism; h: tryptophan metabolism.

The following criteria were used to screen differential metabolites: a fold change of greater than 1.2, a variable importance for the projection (VIP) score greater than 1.0, and a *P* value less than 0.05. The Kyoto Encyclopedia of Genes and Genomes pathway analysis of differential metabolites was performed using MetaboAnalyst. A *P* value <0.05 and an impact >0.2 were used as criteria to screen for metabolic pathways with significant changes. In comparison to the control group, the model group exhibited significant changes in several metabolic pathways, including riboflavin metabolism, histidine metabolism, tryptophan metabolism, arginine biosynthesis, and starch and sucrose metabolism. The metabolic pathways that were significantly changed after H-RLP treatment were phenylalanine, tyrosine, and tryptophan biosynthesis; riboflavin metabolism; tryptophan metabolism; alanine, aspartate, and glutamate metabolism; and phenylalanine metabolism. Among them, riboflavin metabolism and tryptophan metabolism were the common metabolic pathways among the control group, model group, and H-RLP group (Figures 4(a) and 4(b)). By comparing the differential metabolites among the control, model, and H-RLP groups, we identified common differential metabolites. These metabolites, along with the metabolites associated with the significantly altered metabolic pathways, resulted in a total of 22 differential metabolites (Table 1).

**Figure 5:**
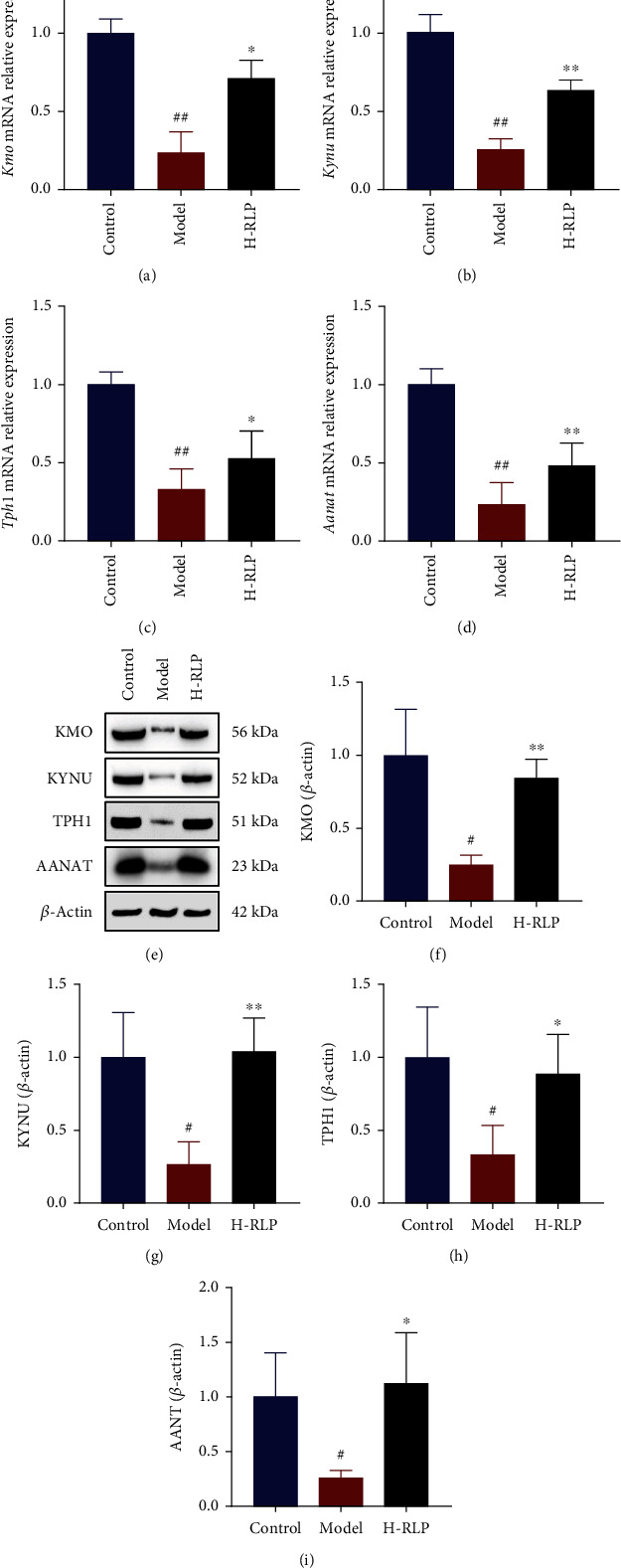
RLP alters tryptophan metabolism-related enzymes in the kidney of DN mice. (a–d) RT-qPCR analysis showed that the decreased mRNA levels of key enzymes involved in tryptophan metabolism in kidney of DN mice, such as *Kmo*, *Kynu*, *Tph1*, and *Aanat*, were improved by RLP treatment. (e–i) Western blot analysis confirmed the increase of KMO, KYNU, TPH1, and AANAT in the kidney of DN mice after RLP treatment.

In the model groups, the levels of key enzymes of tryptophan metabolisms, kynurenine 3-monooxygenase (KMO), kynureninase (KYNU), tryptophan hydroxylase 1 (TPH1), and aralkylamine N-acetyltransferase (AANAT), were decreased compared to the control group. After RLP treatment, the levels of all 4 enzymes elevated in different degrees (Figures 5(a)–5(d)). The levels of mRNA corresponding to the key enzymes were also examined, and the results were consistent with those of western blot (Figures 5(e)–5(i)).

### 3.4. Effects of RLP on Ferroptosis in the Kidney Tissues of DN Mice

**Figure 6:**
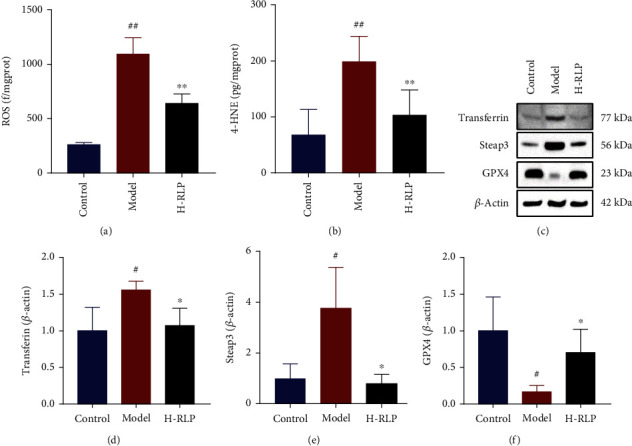
RLP ameliorates ferroptosis in the kidney of DN mice. The elevated levels of ROS (a) and 4-HNE (b) in the kidney of DN mice were significantly decreased after RLP treatment, which indicated the accumulation of a large amount of lipid peroxides in kidney were scavenged. (c–f) RLP decreased the levels of transferrin and Steap3 while increased the level of GPX4, suggesting that both iron overload and GPX4 depletion have been alleviated.

Ferroptosis, a relatively new and intriguing mode of cell apoptosis, has emerged as a distinct process distinguished by the iron-dependent accumulation of lipid peroxides. It represents a novel and intricate pathway involved in cellular demise with unique implications for various physiological and pathological conditions. Studies have shown that ferroptosis is closely related to the onset of DN and that DN may be alleviated by inhibiting ferroptosis of renal tubular epithelial cells [17]. The latest research has indicated that 3-hydroxyanthranilic acid (3-HA), a metabolite of tryptophan, can inhibit ferroptosis. Interestingly, we also found that RLP increased the 3-HA level [18]. Therefore, the effects of RLP on ferroptosis-related factors of kidney tissues in DN mice were investigated. Compared with that observed in the control group, the ROS and 4- HNE levels in kidney of mice in the model group increased, further indicating accumulation of a large amount of lipid peroxides in the kidneys (Figures 6(a) and 6(b)). After H-RLP treatment, ROS and 4-HNE levels decreased significantly, suggesting that lipid peroxidation was cleared. In addition, western blot was performed to detect the levels of ferroptosis-related factors in the kidney of mice. In the kidney of DN mice, there was a notable increase in the relative levels of transferrin and Steap3 proteins. This observation suggests the occurrence of iron overload in the kidney. Conversely, the glutathione peroxidase 4 (GPX4) level was significantly decreased, indicating that GPX4 was depleted (Figures 6(c)–6(f)). After H-RLP treatment, the relative levels of transferrin and Steap3 proteins were decreased, while the protein level of GPX4 was increased, suggesting that both iron overload and GPX4 depletion were alleviated.

### 3.5. Effects of RLP on the PI3K/AKT Pathway and Apoptosis in the Kidney Tissue of DN Mice

**Figure 7:**
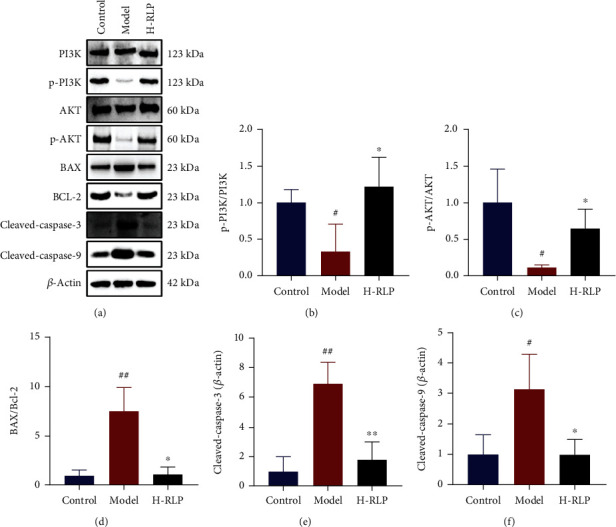
RLP alters the PI3K/AKT signalling pathway and apoptosis in the kidney of DN mice. (a–c) RLP treatment increased the phosphorylation levels of PI3K and AKT in the kidney of DN mice. (d–f) Elevated levels of apoptosis-related proteins BAX, BCL-2, cleaved-caspase-9, and cleaved-caspase-3 were decreased after RLP treatment, suggesting the apoptosis was ameliorated.

Interestingly, the metabolomics results revealed an improvement in the levels of N-acetylserotonin (NAS) following H-RLP treatment. NAS, as a metabolite of tryptophan, has the potential to inhibit apoptosis through the PI3K/AKT pathway [19]. Thus, the effects of RLP on the PI3K/AKT pathway and apoptosis in the kidney tissues of DN mice were investigated in this study. The western blot results demonstrated that the phosphorylation levels of PI3K and AKT were elevated in the model group compared to the control group. Additionally, the protein levels of B-cell lymphoma 2 (BCL-2), BCL-2-associated X (BAX), cleaved caspase-3, and cleaved caspase-9 were increased, indicating severe apoptosis in the kidney tissues that was possibly mediated by the PI3K/AKT pathway. Following H-RLP treatment, the phosphorylation levels of PI3K and AKT were reduced, indicating inhibition of the PI3K/AKT pathway. Moreover, the relative protein levels of BAX, BCL-2, cleaved-caspase-9, and cleaved-caspase-3 were also decreased, suggesting a decrease in apoptosis in the kidney. This suggested that RLP significantly alleviated apoptosis in the kidney tissues of DN mice and that this may be dependent on the PI3K/AKT pathway (Figures 7(a)–7(f)).

## 4. Discussions

In this study, HFD was used in combination with STZ to establish a mouse model of DN, which is a classic DN model that can replicate symptoms similar to DN in humans [20]. The 24 h protein level in urine is the most sensitive indicator for assessing early DN and is usually included in the first step of screening and diagnosis, as it directly reflects impairment of glomerular filtration [21]. Cr and BUN are mainly metabolized and excreted out of the body through glomerular filtration, and their levels generally increase with the progression of DN. The increase in serum Cr and BUN levels indicates the decline of renal function [22]. The model in this study was able to reproduce these symptoms. Moreover, pathological manifestations of the glomerular and tubular epithelial cell hypertrophy as well as thickening of glomerular and tubular basement membrane were observed in the kidney tissues of DN mice. In this study, RLP treatment improved the above-mentioned symptoms of DN. In addition, IRB, a commonly used clinical drug for treating DN, was used as a positive control in this study [23]. Interestingly, no significant difference was observed in the efficacy between H-RLP and IRB groups, indicating that RLP was equally effective as IRB in the treatment of DN. This finding further supports the significant therapeutic potential of RLP in the treatment of DN.

Both oxidative stress and inflammation play pivotal roles in the development and progression of DN. In diabetes, elevated levels of inflammatory cytokines can contribute to increased oxidative stress, leading to further kidney damage. This creates a vicious cycle where oxidative stress and inflammation exacerbate each other, amplifying the pathological processes in DN [24]. Enhanced ROS production has been associated with vasoconstriction, growth and migration of vascular smooth muscle cells, endothelial dysfunction, alterations in extracellular matrix (ECM) proteins, and increased renal sodium reabsorption [25, 26]. Increased oxidative stress in individuals with diabetes is a significant contributor to the development and progression of DN [27]. SOD and GSH-Px are important antioxidants. Therefore, enhancing the activity of SOD and GSH-Px may help reduce oxidative stress and thereby alleviate DN [28, 29]. The impact of inflammatory cytokines on kidney tissues has been associated with factors such expression of different molecules, abnormal hemodynamics in the glomerulus, changes in the ECM and glomerular basement membrane, apoptosis, necrosis, endothelial permeability, and oxidative stress [30–32]. Previous research has consistently demonstrated elevated levels of proinflammatory cytokines, including IL-1*β*, IL-6, and TNF-*α*, in patients with DN, and anti-inflammatory therapy is one of the important therapeutic approaches for treating DN [33]. In the present study, RLP had a significant effect on improving oxidative stress and inflammation, which may be a potential mechanism of RLP in the treatment of DN.

Metabolomic results showed that riboflavin metabolism and tryptophan metabolism were common metabolic pathways among the control, model, and H-RLP groups, suggesting that these pathways may be potential pathways through which RLP improves DN. Vitamin B2, also known as riboflavin, is a core component of riboflavin metabolism. Riboflavin-5′-phosphate (FMN) is a downstream product of riboflavin, and the two may be transformed into each other. Previous studies have demonstrated the beneficial effects of riboflavin supplementation in diabetic mice. These studies have shown that riboflavin treatment leads to a decrease in indicators of the kidney and liver dysfunction, suggesting an improvement in organ function. Additionally, riboflavin has been found to play a role in repairing damaged cellular DNA, which is often a consequence of oxidative stress. By reducing inflammation caused by oxidative stress, riboflavin treatment has the potential to lower the risk of diabetic complications. [34]. In this study, the riboflavin level in the kidney of DN mice was lower than that of normal ones, while the FMN level was higher. RLP treatment significantly corrected the disturbance of riboflavin metabolism, which may be related to the mechanisms for reducing renal injury. However, very few studies have investigated riboflavin and diabetes, and more research is still required to elucidate the underlying mechanisms.

Tryptophan metabolism involves several pathways, including the kynurenine pathway, serotonin (5-HT) pathway, and indole pathway. These pathways are responsible for the breakdown and conversion of tryptophan into various metabolites, which collectively contribute to the overall metabolism of tryptophan and play important roles in various physiological processes. Among them, L-kynurenine and 3-HA are important metabolites in the kynurenine pathway. 5-Hydroxytryptophan (5-HTP) and NAS are important metabolites of the 5-HT pathway, while 2-(1H-indol-3-yl) acetic acid is a metabolite of the indole pathway. Kynurenine and its metabolites 3-hydroxy-L-kynurenine (3-HK) and 3-HA have significant antiferroptosis effects [35]. In the kynurenine pathway, kynurenine is catalyzed by KMO to produce 3-HK, which is catalyzed by KYNU to produce 3-HA. Studies have found that 3-HA is an effective free radical-scavenging antioxidant that can significantly alleviate ferroptosis. KYNU is essential for the synthesis of 3-HA. Knockout of KYNU significantly reduced the 3-HA level and increased the precursor 3-HK level, while sensitizing cells to ferroptosis [18]. Many studies have confirmed that ferroptosis is involved in the development of DN and that inhibition of ferroptosis may delay the development of DN. Moreover, some drugs have been found to improve DN by regulating ferroptosis [5, 17, 36]. 5-HT and its downstream metabolites play important roles in anti-inflammation and antioxidation [37]. Tryptophan is catalyzed by TPH to produce 5-HTP, which is catalyzed by dopa decarboxylase (DDC) to produce 5-HT and then catalyzed by AANAT to produce NAS. NAS has been shown to exhibit protective effects against H_2_O_2_-induced apoptosis and oxidative stress in cells. It achieves this by inhibiting ROS activity and activating the PI3K/AKT signaling pathway [19]. Studies have also found that NAS improved cell damage by inhibiting ferroptosis and that this is achieved at least in part by activating the PI3K/Akt/Nrf2/Fth pathway [38]. 2-(1H-indol-3-yl) acetic acid can attenuate the inflammatory response of lipid-loaded cells, and such responses are aryl hydrocarbon receptor-dependent [39]. However, we could not find more studies that associate 2-(1H-indol-3-yl) acetic acid with DN, indicating a potential future research direction. In this study, extensive disruption of tryptophan metabolism was observed in the kidney tissues of DN mice. Kynurenine, 3-HA, 5-HTP, and NAS levels were decreased to varying degrees. Kynurenine and 3-HA were extensively depleted. In addition, the detection of key enzymes of differential metabolites revealed that the levels of key enzymes and the corresponding mRNA levels were significantly reduced, which may be related to the reduction in the levels of the differential metabolites in tryptophan metabolism. Interestingly, the treatment with RLP showed significant improvements in the levels of these metabolites associated with tryptophan metabolism. Furthermore, RLP administration also increased the levels of key enzymes involved in tryptophan metabolism as well as their mRNA expressions. These findings indicate a potential connection between the therapeutic effect of RLP and the modulation of tryptophan metabolism. It suggests that RLP may exert its beneficial effects by influencing the metabolic pathways associated with tryptophan, highlighting the importance of tryptophan metabolism in the mechanism of action of RLP.

Given the important relationship between tryptophan metabolism and ferroptosis, we subsequently investigated whether RLP could affect ferroptosis in DN mice. The essence of ferroptosis is the excessive accumulation of lipid peroxides caused by the inactivation of GPX4, eventually leading to cell death [40]. In normal physiological conditions, iron ions combined with transferrin are transported into cells and then reduced to ferrous ions with the catalysis by Steap3 to produce a labile iron pool in cells. Once iron metabolism is disrupted, abnormal accumulation of intracellular iron would lead to iron overload. Subsequently, the Fenton reaction would occur to generate a large amount of ROS, subsequently triggering lipid peroxidation [41]. GPX4 is the only enzyme known to scavenge lipid peroxides [42]. When GPX4 is inactivated, the accumulation of lipid peroxides that cannot be cleared in time would eventually trigger cell death. Both in vivo and in vitro studies have found that inhibition of ferroptosis exerts a protective effect on DN [43, 44]. In the present study, RLP reduced the elevated ROS and 4-HNE levels in the kidney tissues of DN mice, thereby alleviating lipid peroxidation. Meanwhile, the levels of transferrin and Steap3 decreased, suggesting that iron overload in the kidney cells was controlled. These findings suggest that RLP can relieve DN by improving ferroptosis.

Furthermore, since NAS, a tryptophan metabolite, has been reported to inhibit apoptosis through the PI3K/AKT pathway, we also investigated whether RLP could affect the PI3K/AKT pathway and apoptosis in DN mice [19]. Apoptosis is also one of the important pathological manifestations of DN. Studies have identified drugs that can inhibit apoptosis by regulating the PI3K/AKT pathway, thereby alleviating DN [45, 46]. PI3K, as a well-known downstream regulatory factor of receptor tyrosine kinases and G protein-coupled receptors, plays a crucial role in various signal transduction pathways under normal physiological and pathological conditions [47]. Phosphatidylinositol (3,4,5)-triphosphate (PIP3), which is produced with the assistance of PI3K, binds to AKT and phosphorylates AKT, ultimately affecting cellular activities, including apoptosis [48]. Studies have found that phosphorylated AKT can regulate BAX/BCL-2, thereby indirectly regulating apoptosis [19]. The BCL-2 protein family is a key factor in apoptosis, and the most important members are BAX, which promotes apoptosis, and BCL-2, which inhibits apoptosis [49]. When BCL-2 expression decreases and BAX expression increases, caspase 9 and caspase 3 would be activated in sequence, which would eventually trigger apoptosis [50]. The findings from this study revealed that RLP intervention had a significant impact on the activation of the PI3K/AKT pathway and the inhibition of renal tissue apoptosis.

**Figure 8:**
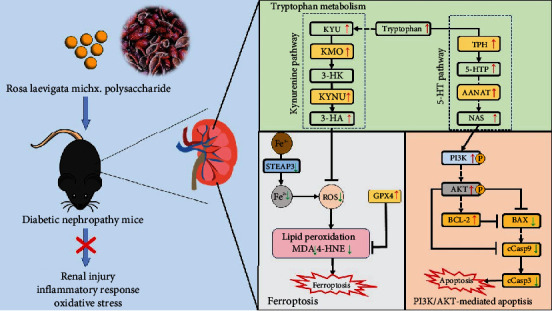
Overview of the main finding of this study.

In conclusion, the findings of this study provide substantial evidence to support the significant therapeutic effect of RLP in STZ-induced DN mice. Metabolomics results showed that the mechanisms of RLP may be related to riboflavin metabolism and tryptophan metabolism. Additionally, RLP demonstrated a remarkable ability to ameliorate ferroptosis and counteract GPX4 inactivation in the kidney tissues of the mouse models employed in this study. Moreover, the protective effect of RLP against apoptosis in the kidney tissues was mediated, at least in part, by the regulation of the PI3K/AKT pathway. These findings shed light on potential underlying mechanisms through which RLP exerts its beneficial effects in improving DN (Figure 8). Further research is warranted to explore and validate these mechanisms, ultimately paving the way for the development of novel therapeutic strategies for DN management.

## Figures and Tables

**Table 1:** Common differential metabolites in the kidney after crocin treatment.

No.	Formula	RT	*m*/*z*	Metabolites	VIP		FC		Trend		Pathway
					M vs. C	R vs. M	M vs. C	R vs. M	M vs. C	R vs. M	
1	C_10_H_9_NO_2_	5.67	174.06	2-(1H-indol-3-yl)acetic acid	1.35	1.07	1.65	0.75	↑#	↓	h
2	C_7_H_7_NO_3_	3.31	154.05	3-Hydroxyanthranilic acid	0.97	1.18	0.26	2.47	↓##	↑^∗∗^	h
3	C_11_H_12_N_2_O_3_	6.55	221.09	5-Hydroxytryptophan	1.63	1.13	0.63	1.52	↓##	↑^∗∗^	h
4	C_4_H_8_N_2_O_3_	1.24	131.05	Asparagine	1.17	1.20	0.83	1.36	↓	↑^∗^	a
5	C_9_H_14_N_4_O_3_	1.67	225.10	Carnosine	1.36	1.08	0.31	7.47	↓#	↑	c
6	C_6_H_8_O_7_	6.00	191.02	Citric acid	1.43	1.28	1.10	1.48	↑	↑^∗^	a
7	C_6_H_13_N_3_O_3_	1.31	174.09	Citrulline	1.33	0.82	1.63	0.78	↑#	↓	b
8	C_6_H_14_NO_8_P	1.20	258.04	D-Glucosamine 6-phosphate	1.84	1.51	1.54	0.43	↑	↓^∗∗^	a
9	C_4_H_4_O_4_	1.20	115.00	Fumaric acid	0.62	1.24	1.72	0.61	↑##	↓^∗∗^	a
10	C_6_H_13_O_9_P	1.52	261.04	Glucose 1-phosphate	1.17	0.98	0.49	1.81	↓#	↑	g
11	C_4_H_7_NO_4_	1.21	132.03	L-Aspartic acid	1.12	1.70	1.17	0.55	↑	↓^∗^	a
12	C_5_H_10_N_2_O_3_	1.32	147.08	L-Glutamine	1.18	1.19	0.69	1.51	↓#	↑^∗∗^	a, b
13	C_6_H_9_N_3_O_2_	1.65	154.06	L-Histidine	1.41	0.38	0.57	1.19	↓#	↑	c
14	C_10_H_12_N_2_O_3_	8.39	209.09	L-Kynurenine	0.69	1.40	0.15	9.61	↓##	↑^∗∗^	h
15	C_11_H_12_N_2_O_2_	6.70	205.10	L-Tryptophan	1.52	1.65	0.60	1.69	↓#	↑^∗^	h
16	C_9_H_11_NO_3_	1.98	180.07	L-Tyrosine	0.40	1.17	0.61	1.57	↓##	↑^∗^	d, e
17	C_7_H_14_N_2_O_3_	1.53	175.11	N-Acetylornithine	1.24	1.15	2.46	0.48	↑##	↓^∗^	b
18	C_12_H_14_N_2_O_2_	7.25	219.11	N-Acetylserotonin	1.22	1.47	0.58	1.39	↓##	↑^∗∗^	h
19	C_9_H_8_O_3_	1.98	163.04	Phenylpyruvic acid	0.81	1.17	0.58	1.61	↓##	↑^∗^	d, e
20	C_17_H_21_N_4_O_9_P	9.46	455.10	Riboflavin-5-phosphate	1.18	0.96	2.22	0.48	↑#	↓	f
21	C_9_H_10_O_5_	7.81	197.05	Syringic acid	1.28	0.80	1.57	0.80	↑#	↓	g
22	C_17_H_20_N_4_O_6_	7.92	377.15	Vitamin B2	1.44	1.41	0.39	2.03	↓##	↑^∗^	f

C: control group; M: model group; R: RLP group; *n* = 6 per group. Name of pathways. a: alanine, aspartate and glutamate metabolism; b: arginine biosynthesis; c: histidine metabolism; d: phenylalanine metabolism; e: phenylalanine, tyrosine, and tryptophan biosynthesis; f: riboflavin metabolism; g: starch and sucrose metabolism; h: tryptophan metabolism. #*P* < 0.05, compared with the control group; ##*P* < 0.01, compared with the control group; ^∗^*P* < 0.05, compared with the model group; ^∗∗^*P* < 0.01, compared with the model group.

## Data Availability

The datasets used and/or analysed during the current study are available from the corresponding author on reasonable request.

## Supplementary Materials

Supplementary Materials The details of materials, reagents, primers, and metabolomic analysis protocol.
