# Supplementary material for: Rosa laevigata Michx. Polysaccharide Ameliorates Diabetic Nephropathy in Mice through Inhibiting Ferroptosis and PI3K/AKT Pathway-Mediated Apoptosis and Modulating Tryptophan Metabolism
Source: J Diabetes Res. 2023 Oct 5;2023:9164883. doi: 10.1155/2023/9164883 (PMC10569897; doi:10.1155/2023/9164883)
Supplement: Supplementary Materials — The details of materials, reagents, primers, and metabolomic analysis protocol. [file 9164883.f1.docx]

**Reagents**

*Rosa laevigata* Michx. polysaccharide (WKQ-0031946) was purchased from Weikeqi biotechnology Co., Ltd. Streptozotocin (S17049) and irbesartan (S42406) were purchased from Shanghai yuanye Bio-Technology Co., Ltd. Assay kits for total protein, creatinine (Cr, C011-2-1) and blood urea nitrogen (BUN, C013-1-1), oxidative stress-related markers superoxide dismutase (SOD, A001-1-2) and glutathione peroxidase (GSH-Px, A005-1-2) activities, and malondialdehyde (MDA, A003-4-1) were purchased from Nanjing Jiancheng Biological Engineering Institute. ELISA kits for Interleukin (IL) -6 (ml063159), IL-1β (ml301814), and tumor necrosis factor α (TNF-α, ml002095) were purchased from Shanghai Enzyme-linked Biotechnology Co., Ltd. Primary antibodies for KMO (ab233529), AANAT (ab3505), transferrin (ab278498), Steap3 (ab104654), GPX4 (ab125066), and ACTB (ab6276), and secondary antibody goat anti-rabbit IgG H&L (ab205718) were purchased from Abcam. Primary antibodies for KYNU (MA5-29723), TPH1 (MA5-32209) were purchased from Invitrogen. Primary antibodies for PI3K (4292), p-PI3K (4228), AKT (9272), p-AKT (86758), BAX (14796), BCL-2 (3498), Cleaved-caspase-3 (9664), Cleaved-caspase-9 (9509) were purchased from Cell Signalling Technique Co, Ltd.

**Table S1 Primer sequence**

| **Gene name** | **Organism** | **Primer sequence (5’-3’)** | |
| --- | --- | --- | --- |
| *Actb* | *Mus musculus* | Forward | CCCCTGAACCCTAAGGCCA |
|  |  | Reverse | ATGGCTACGTACATGGCTGG |
| *Kmo* | *Mus musculus* | Forward | GACTGCCGTGGAGTCCTATG |
|  |  | Reverse | GCACTGTGAGTACCCCTTCC |
| *Kynu* | *Mus musculus* | Forward | ACAAGGGGCCGATTGTGAAT |
|  |  | Reverse | GATGCCATCTGGTTCTCGCT |
| *Tph1* | *Mus musculus* | Forward | TCCCCTCTACACTCCAGAGC |
|  |  | Reverse | CTGGGTTCAGCCAAGAGAGG |
| *Aanat* | *Mus musculus* | Forward | GCTGTGGGACAAGGAGAGAC |
|  |  | Reverse | GCCCAGGTGGTGAAGGTATC |

**Detailed methods of nontargeted metabolomic analysis**

Chromatographic conditions

Chromatographic column: Hypesil Gold column (C18)

Column temperature: 40℃

Flow rate: 0.2 mL/min

Positive mode: Mobile phase A: 0.1% formic acid

Mobile phase B: methanol

Negative mode: Mobile phase A: 5 mM ammonium acetate, pH 9.0

Mobile phase B: methanol

Chromatographic gradient elution procedure

| Time | A% | B% |
| --- | --- | --- |
| 0 | 98 | 2 |
| 1.5 | 98 | 2 |
| 3 | 15 | 85 |
| 10 | 0 | 100 |
| 10.1 | 98 | 2 |
| 11 | 98 | 2 |
| 12 | 98 | 2 |

Mass spectrometry conditions

The scanning range was m/z 100–1500; the ESI source was set as follows: Spray voltage: 3.5kV; Sheath gas flow rate: 35psi; Auxiliary gas flow rate 10L/min; capillary temperature: 320°C; S-lens RF level: 60; Aux gas heater temp: 350°C; Polarity: positive and negative; MS/MS secondary scans: data-dependent scans.

Preparation of blank samples, QC samples, and QC matrix

The blank samples were replaced by 53% aqueous methanol solution, and the pretreatment procedure was the same as that for the experimental samples.
QC samples comprised equal volumes of all experimental samples that were mixed and tested on the machine before, during, and after the liquid chromatography with tandem mass spectrometry injection of the experimental samples.
Before loading the samples, three blank samples and six QC samples were used for testing, of which the first three QC samples were used to monitor the instrument status and equilibrate the chromatography–mass spectrometry system before loading the samples. The next three QCs were scanned in stages and used for metabolite characterization together with the secondary spectra obtained from the experimental samples. After every six samples were tested, one QC was inserted to evaluate the system stability throughout the experiment and to perform data QC analysis.

Metabolite identification

The raw data (.raw) files were imported into CD 3.1 library search software for processing, and simple screening of parameters, such as retention time and mass-to-charge ratio, was performed for each metabolite. Subsequently, retention time deviation of 0.2 min and mass deviation of 5 ppm were set for peak alignment of different samples to make identification more accurate. This step was followed by peak extraction based on information such as mass deviation of 5 ppm, signal intensity deviation of 30%, signal-to-noise ratio of 3, minimum signal strength, and adduct ions while the peak area was quantified; the target ions were then integrated. The molecular formula was predicted with molecular ion peaks and fragment ions and compared using mzCloud (<https://www.mzcloud.org/>), mzVault, and Masslist databases, and the blank samples were used to remove the background ions. The raw quantification results were normalized to obtain the metabolite identification and relative quantification results. Data processing was based on the Linux operating system (CentOS version 6.6) and the software R and Python.

Data analysis

Metabolites identified using the KEGG database (<https://www.genome.jp/kegg/pathway.html>) were annotated.
In multivariate statistical analysis, the data were transformed using the metabolomics data processing software metaX and then subjected to PCA and partial least squares discriminant analysis (PLS-DA) to obtain variable importance projection (VIP) values for each metabolite. In univariate analysis, statistical significance (P value) was calculated for each metabolite between the two groups based on t-test, and the FC value of the difference between the metabolites between the two groups was calculated. The criteria for differential metabolite screening were VIP > 1, *P* < 0.05, and FC ≥ 1.2 or FC ≤ 0.83.
